# Pig Sedation and Anesthesia for Medical Research

**DOI:** 10.3390/ani13243807

**Published:** 2023-12-10

**Authors:** Ruxandra Costea, Ioana Ene, Ruxandra Pavel

**Affiliations:** Faculty of Veterinary Medicine, University of Agronomic Sciences and Veterinary Medicine, 011464 Bucharest, Romania

**Keywords:** sedation, anesthesia, pig, research models, protocols

## Abstract

**Simple Summary:**

Anesthesia plays a crucial role in ensuring the ethical treatment of research animals and obtaining reliable and accurate data. Pig anesthesia is a significant aspect of clinical veterinary practice, especially when performing surgical procedures, diagnostic imaging, various medical interventions, and scientific research procedures. Proper anesthesia protocols ensure that the animals are kept unconscious and do not experience pain or distress, which is not only ethically responsible but also needed by regulatory bodies and animal welfare standards. This article is a narrative review that presents considerations for sedation and anesthesia of pigs, highlighting species particularities and reviewing the agents and protocols commonly used for medical and scientific research.

**Abstract:**

In clinical veterinary practice, proper training and expertise in anesthesia administration and monitoring are essential. Pigs are suitable experimental animals for many surgical techniques because they are similar in size to humans and have a short reproductive cycle. This makes them ideal for research concerning organ transplantation, cardiovascular surgery, and other procedures that require a large animal model. Sedation and premedication should be administered at the lowest dose to be effective with predictable results and reduced adverse effects, to ensure the safety of both the animal and the team involved in the procedure, with a fast onset and optimizing the induction and maintenance of anesthesia. The goal of induction is to achieve a safe and effective level of anesthesia that ensures patient safety and facilitates research. Most of the time, inhalation anesthesia with endotracheal intubation is the ideal choice for maintenance of anesthesia. The difficulties related to endotracheal intubation of pigs can be overcome by knowing the anatomical peculiarities. Effective analgesia tailored to the specific procedure, the pig’s condition, and individual responses to medications should complete the maintenance and recovery protocols, reducing perioperative complications.

## 1. Physical Examination

Anesthesia ensures the welfare of the animal, enables safe and effective procedures, and allows accurate data collection [1]. Pigs are commonly used in medical and scientific research as models for studying various aspects of human health, physiology, and disease due to their physiological and anatomical similarities to humans [2,3,4,5]. Pigs are known to be highly sensitive to stress; consequently, they should be conditioned at the research facility for approximately 7–14 days before anesthesia, in order to have time to adapt to the experimental environment, to avoid stress-induced respiratory disease or diarrhea [6,7].

Physical preanesthetic examination must be performed in a low-stress environment with a focus on evaluation of respiratory and cardiovascular system function. Age and maturity criteria should be considered when choosing a model. The majority of pigs utilized in research projects weigh 15 to 30 kg and are 8 to 12 weeks old [6]. The decision to withhold food and water preoperatively in pigs should involve consideration of the animals’ age, growth rate, breed, pregnancy status, clinical status, and the procedure to be performed. Food and water withdrawal regimens have a wide variation of 2–12 h, with particularly aggressive fasting regimes for gastrointestinal or abdominal surgery [8]. Although fasting may reduce the risk of regurgitation, fasting is recommended, as aspiration of regurgitated material can occur and may cause airway obstruction, irritation, and ultimately aspiration pneumonia. Aspiration of acidic stomach fluid may cause immediate reflexive airway closure and destruction of type II alveolar cells and pulmonary capillary lining cells. Consequently, pulmonary edema and hemorrhage may develop along with bronchospasm, dyspnea, hypoxemia, and cyanosis. Recovery from aspiration pneumonia, which may take a few days to develop, depends on the pH of the material aspirated. Swine tend to have very acidic stomach fluid with a pH as low as 1.5–2.5 [9,10]. Alfalfa and other types of hay can delay gastric emptying time, which means that vomiting and aspiration may still occur even after a 12-h fasting period. To avoid this, alfalfa or other forms of hay should be eliminated from the regular diet 2–3 days before general anesthesia [11]. Piglets, who are prone to hypoglycemia, should be denied suckling for only 1–2 h before anesthetic induction [9]. 

Following the preanesthetic physical examination, pigs can be included in a corresponding anesthetic risk classification system according to the American Society of Anesthesiologists (ASA) physical status classification system modified for veterinary medicine, which is a valuable prognostic tool, recommended to identify an increased risk of anesthetic complications and mortality [12,13].

## 2. Recommendations for Injectable Administration

**Figure 1:**
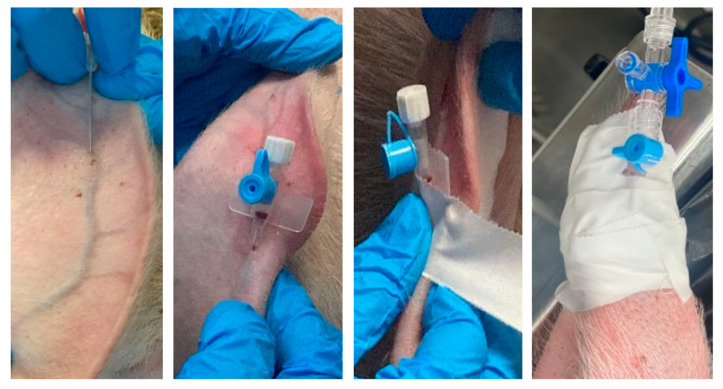
Mounting and fixing a peripheral catheter in the auricular vein.

Injections should be performed slowly, if possible, to minimize pain associated with injection and tissue damage [13]. The dimensions of the needle must be selected with consideration of the size of the animal and the liquid consistency of the injectate (aqueous or oily). For subcutaneous (SC) or intramuscular injections (IM), an extension line can be used to connect the syringe and cannula to reduce the risk associated with any evasive movements of the pig [14]. As the skin of swine can only be tented to a minor degree, only small-volume SC injections can be delivered [15]. Two locations are suitable for SC injections and are recommended: the knee fold (body weight under 20 kg) or caudal to the ear base for larger pigs [14,16]. The muscles of the caudal thigh region, semimembranosus and semitendinosus, and the gluteal muscles of the cranial thigh are generally selected as suitable sites for large-volume intramuscular injections (IM), while for small volumes to be injected, it is preferred to access the dorsolateral neck region. The injection can be performed in a less stressful way for the pig if it is possible to feed it simultaneously [16,17]. Intravenous access (IV) can be challenging because pigs resist restraint and they have very few superficial veins accessible for IV injection or catheterization [9,17]. The auricular veins, jugular vein, and femoral vein are all commonly used for drawing blood or administering fluids in pigs. The auricular veins located on the lateral and medial dorsal ear margins offer the easiest access for intravenous injection [18,19]. Topical application of a eutectic mixture of lidocaine 2.5% and prilocaine 2.5% for anesthesia has been used for various procedures in human medicine and although studies in animals are limited, it appears to facilitate various procedures in veterinary medicine, including venipuncture [20]. Puncture of the ear vein requires physical restraint of the swine or heavy sedation. After occluding blood flow at the base of the ear, the vessels are easy to identify (Figure 1). Catheterization of the jugular or femoral veins can be challenging and should only be performed by experienced personnel [14,15]. 

## 3. Sedation and Premedication

Sedation is often suitable for minor procedures, such as physical examination and diagnostic imaging. or it represents premedication for anesthesia. The choice of an appropriate sedative protocol should be based on the procedure’s type, animal health status, age, and size. Other factors, such as the desired level of sedation and the duration of the procedure, influence the selection of medication. Sedatives should be administered at the lowest effective dose to minimize the risk of adverse effects and calculated based on the pig’s weight. After sedation, pigs may still need some level of physical restraint to ensure the safety of both the animal and the people involved in the procedure [12,15]. 

Stress during handling and restraint can lead to increased vocalizations, making the process of injection of sedative drugs challenging. Small pigs (<10 kg) may be more easily restrained compared to larger ones and less prone to stress-related vocalizations [1].

Multiple classes of agents may be considered for sedation in pigs. A detailed chart of dosage, route, and other considerations is listed in Table 1. Short, minimally invasive procedures may require lighter sedation with a focus on anxiolysis, achieved through benzodiazepines and alpha-2 adrenoreceptor agonists. Some examples of sedation protocols include azaperone, acepromazine, diazepam, midazolam, xylazine, and medetomidine, used alone or in combination. For more invasive surgeries, a combination of sedatives, analgesics, and anesthetics may be employed to ensure deep sedation, pain control, and a stable anesthetic plane. When deeper sedation is necessary, ketamine can be added to the combinations. The combination of tiletamine and zolazepam produces heavy sedation and immobilization with a relatively small volume of injection, making it particularly suitable for larger animals [1]. If pain is present or anticipated for the procedure, the protocols may also include opioids such as buprenorphine, morphine, or methadone.

Premedication refers to the administration of medications prior to the induction of anesthesia, minimizing stress and anxiety, providing pre-emptive analgesia, and optimizing the induction and maintenance of anesthesia. The ideal premedication agent must be effective with predictable results and fast onset, easy to administer, reversible, and offer analgesia and muscle relaxation with minimum cardiovascular and respiratory depression. Medication and protocols will be decided based on the preanesthetic evaluation (ASA status, temperament, procedure, level of pain expected), anesthetist’s level of experience, and equipment available [13]. 

Protocols for premedication usually include multiple agents, to achieve the maximum effect with minimum secondary effects. The use of the anticholinergics glycopyrrolate and atropine has the potential to reduce salivation and bronchial secretions, but should be performed with caution considering their cardiovascular effects [15]. 

In the authors’ practice, the most common combination for sedative drugs used for pigs includes IM administration of ketamine (10–20 mg/kg), xylazine (1–2 mg/kg), and midazolam (0.1–0.2 mg/kg), with alternative combinations that include medetomidine or dexmedetomidine [12]. The lower doses are usually used for sedation and the higher are intended for anesthetic premedication. 

Detailed considerations regarding the dosage, route of administration, and relevant data for sedation and premedication are shown in Table 1.

### 3.1. Butyrophenones

Azaperone is a neuroleptic sedative medication that belongs to the class of butyrophenone derivatives. It is widely used in pigs to provide sedation, reduce anxiety, calm animals, and combat aggression and stress in pigs [35,36]. Azaperone works at central adrenergic dopamine D2 receptors located in the reticular activating system, leading to its sedative and anti-anxiety effects [37]. Vasodilation, hypotension, and hypothermia may occur following the administration of azaperone so it should not be used in debilitated, hypovolemic, or hypotensive pigs. It can also be used for maiden sows after their first litter to reduce the rejection of piglets [22]. Azaperone given alone by the intramuscular route has a rapid onset of action (5–20 min) with a duration of action of 2–6 h (maximal effects within 30 min), while intravenous injection often results in excitation [9]. Oral or intranasal administration of azaperone at a dose of 4 mg/kg induces sedation in piglets that is clinically comparable to an intramuscular administration of 2 mg/kg [38,39]. Deeper sedation with fewer adverse effects can be achieved by combining azaperone with ketamine and butorphanol [22,29] or azaperone with ketamine and an alpha-2 adrenoreceptor agonist [30,40]. Susceptible Pietrain pigs were protected against halothane-induced malignant hyperthermia with azaperone at doses of 0.5–2 mg/kg IM [11,41].

### 3.2. Phenothiazines

Acepromazine (0.11–1.1 mg/kg IM, IV, SC) is commonly used alone for tranquilization [21]. This drug decreases spontaneous motor activity and may cause hypotension and hypothermia [9]. The recommended dose of 0.1–0.4 mg/kg IV or IM may be used in combination with other drugs to improve the quality of premedication [40]. The combination of acepromazine with ketamine or tiletamine/zolazepam produces reliable sedation and muscle relaxation [36]. Acepromazine 1.1–1.65 mg/kg IM has been reported to reduce the incidence of malignant hyperthermia related to anesthesia [41,42].

### 3.3. Benzodiazepines

Benzodiazepines are a class of sedative and anxiolytic drugs that are commonly used in both human and veterinary medicine. They work by enhancing the effects of a neurotransmitter called gamma-aminobutyric acid (GABA), which leads to sedative, anxiolytic (anti-anxiety), muscle relaxant, and anticonvulsant effects [43]. Midazolam, when compared with diazepam, is water-soluble, is absorbed rapidly, has a higher affinity for receptors, stronger potency, and quicker onset with a shorter duration of effect [9]. Diazepam and midazolam can be used in combination with ketamine, alpha-2 adrenoreceptor agonists, and opioids. When used in combination with ketamine, muscle relaxation will be improved during anesthesia [36], and when used in combination with alfaxalone (5 mg/kg IM), muscle relaxation and sedation levels increase [9]. Intranasal administration of midazolam (0.2 mg/kg) provides reliable sedation (effect in 3–4 min) [44]. Less commonly used benzodiazepines include flurazepam 2 mg/kg IV [43] and lorazepam 0.1 mg/kg [15]. Flumazenil 0.02–0.08 mg/kg is a selective benzodiazepine antagonist reversal agent that can be used to counteract the effects of benzodiazepines in cases of overdose or adverse reactions, or to facilitate recovery from sedation or anesthesia [45].

### 3.4. Alpha-2 Adrenoreceptor Agonists

Alpha-2 adrenoreceptor agonists are a class of medications that activate specific receptors in the body. These medications have various effects, including sedation, analgesia, muscle relaxation, and vasoconstriction. Alpha-2 adrenoreceptor agonists are often used for sedation, preanesthetic medication, and pain management in pigs, alone or as part of a balanced anesthesia protocol in combination with other medication, such as anesthetics and analgesics [46]. Pigs are more resistant to alpha-2 adrenoceptor agonists than ruminants and other domestic animals and require a higher dosage for mild to moderate sedation [46,47]. While alpha-2 agonists have beneficial effects, they can also cause side effects such as bradycardia, decreased respiratory rate, hypotension, decreased gastrointestinal motility, and hypothermia. Reversal agents (e.g., atipamezole, yohimbine, tolazoline, vatinoxan) are available to antagonize the effects of the alpha-2 adrenoceptor agonists [36].

Intramuscular administration of medetomidine at doses ranging from 0.04 to 0.08 mg/kg induced sedation and muscle relaxation, with an increasing effect observed at higher doses [34]. However, increasing the dose above 0.1 mg/kg did not further intensify sedation or muscle relaxation, but instead prolonged the duration of these effects. Medetomidine (0.04 mg/kg IV or 0.08 mg/kg IM) in combination with ketamine has been utilized in pigs for short-term anesthesia [34]. Medetomidine, when combined with butorphanol (0.2 mg/kg IM) and ketamine (10 mg/kg IM), produced prolonged anesthesia in pigs compared to a combination of xylazine (2 mg/kg IM), butorphanol (0.2 mg/kg IM), and ketamine (10 mg/kg IM). The achieved muscle relaxation was adequate for tracheal intubation, but moderate cardiovascular depression was observed after using the combination of medetomidine, butorphanol, and ketamine for anesthesia [48]. In a specific study involving young pigs, the administration of a combination of 0.08 mg/kg medetomidine and 0.2 mg/kg butorphanol did not provide adequate sedation to facilitate blood sampling in all animals [49]. 

### 3.5. Dissociative Anesthetics 

Ketamine is an NMDA (N-methyl D aspartate) receptor antagonist drug that can be used for sedation in pigs. It works by antagonizing the effects of the neurotransmitter glutamate, resulting in sedation, analgesia, and dissociation from the environment. Ketamine is often used in combination with other medications to achieve the desired level of sedation or anesthesia. Ketamine can cause side effects such as increased muscle tone, muscle fasciculations, poor muscle relaxation, and analgesia when used alone [36]. Occasionally, pigs may experience a period of disorientation and ataxia during recovery from ketamine sedation and might need a comfortable environment to prevent injury during this phase. These effects can be managed and minimized through appropriate dosing and the use of ketamine combined with other medications [50]. In healthy animals, ketamine has a good analgesic effect and only slightly modifies heart rate. When ketamine is administered alone, the ability of the swallowing reflex is unaffected, but excitation and excessive salivation can develop during anesthesia and recovery [22]. Tiletamine is a dissociative anesthetic used in veterinary medicine in combination with zolazepam (Telazol^®^ tiletamine/zolazepam) to induce sedation or anesthesia in pigs. Tiletamine is approximately twice as potent as ketamine and has a longer duration of action [51]. Telazol^®^ (tiletamine/zolazepam, 4.4 mg/kg) and xylazine (2.2 mg/kg) IM provide rapid sedation and can be used for sedation and induction [47]. Pigs often experience prolonged and rough recovery characterized by swimming motions, with repeated attempts to right themselves when recovering from Telazol anesthesia, similar to that observed when ketamine is used alone [41,52]. Studies have shown that tiletamine and zolazepam are both eliminated more slowly in pigs than in other species and that tiletamine has a longer effect than zolazepam in pigs [52]. Flumazenil can be used to antagonize zolazepam, but care should be granted to avoid residual effects of tiletamine leading to excitation, muscular tone, and fasciculations [23,45]. 

### 3.6. Opioids

Opioids are a class of medication commonly used for pain management and sedation in pigs, acting by binding to specific receptors in the nervous system (opioid receptors), which results in pain relief, sedation, and other effects [12,37]. Opioids can be used for sedation in pigs, particularly for pain management and calming effects. Opioids can be used in combination with other sedatives, anesthetics, or analgesics to achieve the desired level of sedation and pain control; pure µ agonists result in a strong analgesic effect, and partial μ agonists can be used in protocols for moderate pain along with μ- antagonists/K-agonists. Opioids can cause side effects such as vocalization, excitations, respiratory depression, decreased heart rate, and constipation. Butorphanol, administered at 0.2 mg/kg intramuscularly, resulted in important behavioral changes in piglets, resembling panic attacks, which have not been described in this species before [53]. The administration of buprenorphine did not decrease piglet vocalizations during the castration procedure but proved to be highly successful in mitigating pain behaviors [54]. In the post-surgery recovery, buprenorphine alleviated pain related to different surgical procedures, but had reduced effectiveness in addressing pain symptoms associated with inflammation, organ failure, or systemic disease when compared to pain associated with surgical incisions, orthopedic, dental, or ophthalmic procedures [55]. Buprenorphine has a relatively long duration of effect and low rate of side effects, but doses higher than 0.01 mg/kg must be used bearing in mind a possible respiratory depression [14]. Fentanyl, a short-acting opioid, can be used in pigs as a constant intravenous infusion at rates varying from 10 to 100 µg/kg/h without major side effects [46]. Boluses of morphine and fentanyl infusions will decrease the minimum alveolar concentration (MAC) levels of isoflurane [41]. Fentanyl and buprenorphine can also be used as transdermal patches, providing long-term analgesia, with a reduced incidence of side effects [56]. An example of ensuring preoperative and postoperative analgesia is represented by the protocol consisting of epidural morphine (0.1 mg/kg) prior to abdominal surgery, and a transdermal fentanyl patch (50 mg/h) postoperatively, which contributes to almost immediate restoration of normal activity levels and weight gain after recovery from general anesthesia [57]. Reversal agents available (e.g., antagonist naloxone 0.5–2 mg/kg IV [21]) can counteract negative side effects of opioids and can be used in unexpected reactions or overdose. In these cases, analgesic effects will also be reversed.

### 3.7. Alfaxalone

Alfaxalone is a neurosteroid anesthetic agent used for sedation, induction, and maintenance of anesthesia, with a rapid onset and relatively short duration of action. Alfaxalone can be administered both IV and IM in pigs [58,59]. Alfaxalone can cause side effects such as respiratory depression, decreased heart rate, and a decrease in blood pressure. Alfaxalone has been used in pigs to induce and maintain anesthesia with minimal cardiovascular effects [31,53]. A combination of alfaxalone and dexmedetomidine can be used to maintain long-duration total intravenous anesthesia in pigs [32,60].

### 3.8. Local Anesthetics

Lidocaine and bupivacaine are local anesthetic medications commonly used for various purposes in pigs, including local anesthesia for surgical procedures, postoperative pain management, and nerve blocks [24]. While local anesthetics are generally well-tolerated, some pigs may experience hypersensitivity or allergic reactions to the medications [37]. Careful observation of adverse reactions is important [61]. Lidocaine is widely used intravenously in different species to provide analgesia and as an adjunct to general anesthesia. In one experimental model of lung transplantation, intravenous lidocaine was associated with an attenuation of the histological markers of lung damage in the early stages of reperfusion [62]. Administration of lidocaine may help to prevent lung injury during surgery with one lung ventilation, reducing the expression of proinflammatory cytokines and lung apoptosis [63]. 

### 3.9. Neurokinin-1 (NK-1) Receptor Antagonists—Maropitant

Maropitant is a potent, selective neurokinin (NK-1) receptor antagonist primarily administered before anesthetic premedication (1 mg/kg q 24 h, IM) as an antiemetic medication [64]. The MAC of sevoflurane is decreased by maropitant, indicating a potential role as an adjunct visceral analgesic, as demonstrated in other animals [65]. Thus, there is a potential for future applications for swine.

### 3.10. Non-Depolarizing Neuromuscular Blocking Agents (NMBs)

In biomedical research, the use of non-depolarizing neuromuscular blocking agents (NMBs) involves profound muscle relaxation and prevents accidental awareness in conditions of inadequate anesthesia or analgesia. NMBs are widely recommended for tracheal intubation, which is relatively difficult in swine. Studies are quite controversial regarding the achievement of these objectives [66]. When using NMBs, pigs must be unconscious and controlled ventilation must be used. NMBs are not recommended for routine use or without advanced monitoring, which includes measuring arterial blood pressure and neuromuscular blockade assessment with a peripheral nerve stimulator. The NMBs can be administrated as boluses or continuous-rate infusions. Reversal of the neuromuscular blockade involves administration of an acetylcholinesterase inhibitor (neostigmine, edrophonium), which can also generate side effects such as bradycardia and gastrointestinal stimulation. To reduce parasympathetic stimulation, it is recommended to administer an anticholinergic (atropine, glycopyrrolate) before the antagonization of the NMBs. The most common NMBs used are pancuronium, vecuronium, atracurium, and rocuronium. Although monitoring of neuromuscular blockade is possible in pigs, neuromuscular blockade is rarely objectively monitored and is often administered based on clinical signs such as the return of spontaneous ventilation [67]. 

## 4. Induction of Anesthesia

The induction of anesthesia is the process of administering medication to initiate general anesthesia. Preoxygenation with supplemental oxygen via a mask or a flow-by technique can increase the oxygen concentration in the lungs and bloodstream, reducing the risks for hypoxia during induction. The goal of induction is to achieve an adequate depth of anesthesia to prevent any perception or response to the procedures being performed. Induction agents are administered by the inhaled or intravenous route, or a combination (Table 2), depending on the patient and surgical setting. Inhalational induction is not preferred as a method for the induction of anesthesia in pigs, due to the lack of predictable effects, the high volume of volatile agents necessary, and increased risks for the personnel. Ketamine, thiopental, propofol, and alfaxalone are the drugs most commonly used for inducing anesthesia in pigs, due to their fast-acting effects and short recovery time. Thiopental is a thiobarbiturate used for maintenance of anesthesia with tracheal intubation and positive pressure ventilation, as apnea may occur. Ketamine administration alone is not recommended but it can be combined with propofol for endotracheal intubation [68].

## 5. Endotracheal Intubation

Endotracheal intubation is necessary to protect the airway, preventing aspiration and maintaining positive pressure ventilation during anesthesia [70]. Swine intubation is challenging, technically difficult, and requires experience due to anatomical features: the shape of the head, thick, muscular, long tongue, long and narrow oropharyngeal space, small larynx, and an undersized trachea compared to many other animals. The elongated soft palate can hide the epiglottis and partially obstruct the airway, making breathing more difficult, especially in brachycephalic breeds of pigs [18]. The pharyngeal diverticulum is an anatomical structure found in pigs that protrudes from the wall of the pharynx, above the esophagus. The presence and length of the pharyngeal diverticulum (3–4 cm in adults, 1 cm in piglets), can vary among individuals and affect the ease of intubation [19]. The porcine larynx is tubular and lies caudal to the intermandibular space. The structural elements are divided into the thyroid cartilage, the cricoid cartilage, and some primitive arytenoid cartilage. This organ creates a characteristic obtuse angle with the trachea [19]. This anatomical characteristic, along with the existence of the lateral laryngeal ventricles, or ventricles of Morgagni, has been cited as the cause of the difficulty that may be encountered when intubation is performed [18,19]. The vocal cords are positioned caudoventrally [18] and can be easily traumatized if too much pressure is applied during tracheal intubation [19].

Both dorsal and ventral recumbency are described as positions for endotracheal intubation, but ventral recumbency is crucial in facilitating safe and fast intubation and reduces the risk of airway obstruction determined by overextension of the head [19,22,41]. Ventral recumbency can be advantageous if compared to dorsal, especially for operators lacking experience in anesthetizing animals [71]. To decrease the risk of laryngeal spasm, the arytenoids can be sprayed with 2–4% lidocaine a minute before intubation is attempted [46,72]. 

**Figure 2:**
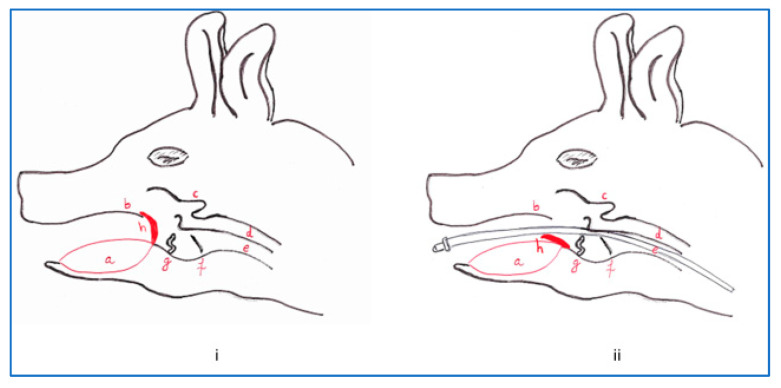
Anatomical features of the oropharyngeal region in pigs (**i**), advancement of the endotracheal tube (**ii**)—a, tongue; b, soft palate; c, pharyngeal diverticulum; d, esophagus; e, trachea; f, lateral ventricle; g, vocal cord; h, epiglottis.

A laryngoscope with a long, straight blade and a plastic guide wire (bougie) can be used to facilitate introduction of the endotracheal tube (ETT) [73]. Some techniques are described using a urinary catheter, a rigid stylet through the tube [41], or a rigid semiflexible intubating stylet adapted manually [12]. The laryngoscope should be introduced until the base of the epiglottis, pressing the tongue followed by lifting the soft palate with the tip of the tube. The ETT is advanced under direct visualization into the trachea (Figure 2). If the ETT cannot be advanced, it should be gently rotated around its longitudinal axis. Straight tubes made of soft material may be advantageous in diminishing the risk of laryngeal trauma. To avoid any aspiration, it is recommended to use cuffed endotracheal tubes and to have available equipment for suction if regurgitation appears. Due to the anatomical particularities in many situations, a flexible connector can be added between the endotracheal tube and the circuit. Ideally, successful and smooth intubation should be performed on the first attempt. If resistance is encountered during intubation at the level of the arytenoid cartilages, a smaller ETT should be used. Repeated attempts during a standard intubation procedure can determine laryngospasm and laryngeal trauma [72,74]. Extubating is performed gently to avoid any traumatization of the tissues; their edema can cause obstructions of the airways during the awakening period. Each time the patient’s position changes, the endotracheal tube must first be disconnected from the respiratory circuit. As an alternative to ETT, a laryngeal mask can be used. The mask is designed to be positioned over the larynx and enable positive pressure ventilation if required [1]. In neonatal piglets, ETT can be very difficult, so the use of a bougie to guide a laryngeal mask during placement can reduce the potential of airway obstruction [75].

## 6. Maintenance of Anesthesia

Maintenance (Table 3) of anesthesia can be performed by administering intravenous anesthetics (total intravenous anesthesia—TIVA), volatile/inhaled anesthetics, or mixed (partial intravenous anesthesia—PIVA) [76,77]. A hypermetabolic response to potent volatile anesthetic gases such as halothane, sevoflurane, desflurane, and isoflurane can trigger malignant hyperthermia, a pharmacogenetic disorder of skeletal muscle [78]. Maintenance of anesthesia can be complimented with a multimodal approach by the use of local anesthesia. Lumbosacral epidural anesthesia is the most commonly used form of regional analgesia in swine [9]. For maintenance of anesthesia, in the authors’ practice [77], the most common PIVA protocol used isoflurane (1–1.5%) in combination with IV infusion of ketamine (1–3 mg/kg/h) and lidocaine (3–6 mg/kg/h). 

Effective analgesia, in a pre-emptive approach tailored to the specific procedure, can prevent the onset of pain and minimize the sensitization of pain pathways, reducing the overall pain experience. Using a combination of different classes of analgesic drugs can provide more comprehensive pain relief [14,79]. Multimodal analgesia involves using opioids, nonsteroidal anti-inflammatory drugs (NSAIDs), local anesthetics, and other pain-relieving medications. NSAIDs are commonly used to reduce inflammation and inhibit pain signaling pathways. NSAIDs alone might not provide sufficient pain control for more invasive procedures, so they should be used in combination with other analgesic medications or techniques [80,81]. The specific choice of NSAIDs and its dosing regimen should be determined by the individual pig’s health status, the procedure being performed, and other relevant factors, to ensure the safety and well-being of the animals. NSAIDs such as meloxicam or flunixin meglumine can help reduce inflammation and provide analgesia [80]. They are particularly useful for managing postoperative pain and are often used in combination with opioids. Local anesthetics such as lidocaine or bupivacaine can be administered via various nerve blocks or wound infiltration to provide targeted pain relief to specific areas and to reduce the need for systemic analgesics and in some cases, continuous infusion of analgesic medications can maintain a consistent level of pain relief throughout the procedure and into the recovery period [82]. Effective pain management should continue into the recovery period and protocols should be adjusted based on the pig’s response and pain level. Crystalloid fluids during anesthesia are used to maintain homeostasis, to cover losses, to restore blood volume, and for stabilization, usually given at a rate of 5–10 mL/kg/h IV. For patients younger than 12 weeks, glucose 5% can be given to prevent hypoglycemia [83].

**Table 3:** Maintenance agents in pigs.

Agent	Dose	Route	Considerations, References
Isoflurane	1.6–1.9% MAC	ETT	[84]
Sevoflurane	2.4–2.66% MAC	ETT	[85]
Propofol	2–3 mg/kg, followed by 0.1–0.2 mg/kg/min	IV	[24]
Alfaxalone	4.8 mg/kg/h	IV	[31]
Fentanyl	50 µg/kg, followed by CRI 30–100 µg/kg/h.	IV	[23,46]
Alfaxalone Dexmedetomidine	5.3 mg/kg/h alfaxalone 3.0 μg/kg/h dexmedetomidine	IV	[32]
Alfaxalone Dexmedetomidine Ketamine	5 mg/kg/h alfaxalone 4 μg/kg/h dexmedetomidine 5 mg/kg/h ketamine	IV	[60]
Medetomidine Butorphanol Ketamine	0.03–0.08 mg/kg medetomidine 0.2 mg/kg butorphanol 10 mg/kg ketamine	IM	Longer sedation than Xylazine-Butorphanol-Ketamine [48]
Xylazine Ketamine Midazolam	2 mg/kg xylazine 0.25 mg/kg midazolam 10–20 mg/kg ketamine	IM	Immobilization in 2 min, effect for 50–90 min [8]
Tiletamine/Zolazepam Telazol^®^ Xylazine	4.4–6 mg/kg tiletamine/zolazepam 2–2.2 mg/kg xylazine	IM	Provides rapid sedation and can be used for sedation and induction [45,47]
Tiletamine/Zolazepam Telazol^®^ Medetomidine	5 mg/kg tiletamine/zolazepam 0.005 mg/kg medetomidine	IM	Provides rapid sedation and can be used for sedation and induction [45,47,56]
Guaifenesin Ketamine Xylazine “Triple drip”	50 mg Guaifenesin 2 mg Ketamine 1 mg Xylazine CRI 2.2 mL/kg/h	IV	Recovery in 30–45 min, Guaifenesin- centrally acting muscle relaxant [23,47]
Flunixin Meglumine	1–4 mg/kg q 24 h.	IV	managing postoperative pain [23]
Meloxicam	0.4 mg/kg	IM	managing postoperative pain [8,22]
Carprofen	1–4 mg/kg q 12 h. 2 mg/kg q 24 h.	IM, IV	managing postoperative pain [8]

## 7. Perianesthetic Monitoring and Complications

Safely managing anesthesia requires a thorough understanding of the indicators linked to the depth of anesthesia and the continuous surveillance of both the patient and the anesthetic apparatus. Monitoring during anesthesia enables evaluation of the depth of anesthesia, adjustment depending on patient particularities, and lastly, the monitoring of body functions during the procedure and in the recovery. Assessing anesthesia depth should be performed every 5–10 min, by evaluation of muscle relaxation, of the jaw tone, absence of movements, and absence of palpebral during anesthesia. If ketamine is included in the anesthetic protocol, ocular reflexes are not reliable [14]. 

For short surgeries, basic monitoring is recommended, while for surgeries that last more than 60 min or with patients who belong to the risk group ASA III-V, additional monitoring is recommended. Basic monitoring should include heart rate, pulse rate and quality, respiratory rate, mucous membrane color, capillary refill time, oxygen saturation, and temperature [40]. The pulse can be detected by feeling the auricular artery, the brachial artery, the saphenous artery, or the sublingual artery on the ventral surface of the tongue. Pulse oximetry measures both pulse rate and the percentage of oxygenated hemoglobin. The probe can be best placed on the pig’s tongue, lip, or ear, but also on the eyelid [40,41], tip of its tail, or in the interdigital space for unpigmented animals. Direct auscultation of the heart should also be performed. In swine, the normal heart rate typically falls within the range of 60 to 90 beats per minute. During anesthesia, drugs such as ketamine and alpha-2 adrenoreceptor agonists can have a significant effect on the heart rate, causing tachycardia or bradycardia, respectively. Rate, rhythm, and pattern of respiration should be assessed during anesthesia. Temperature should be periodically assessed, and appropriate warming methods should be applied during anesthesia in order to prevent hypothermia.

Additional monitoring involves capnography, arterial blood pressure measurement, electrocardiography (ECG), assessment of urinary output, and blood glucose concentration [9]. Capnography analyzes the CO_2_ concentration in the gases expired by the patient and evaluates the adequacy of ventilation, equipment integrity, and the cardiovascular system. ECG monitoring for detecting dysrhythmias can be easily performed in pigs, especially using patch electrodes. Pigs have a prolonged Q-T interval compared to other species [6]. Non-invasive blood pressure measurement is relatively easy in pigs, with either oscillometric or Doppler flow monitors, and a cuff that should be between 40% and 60% of the circumference of the limb [40]. 

If non-depolarizing neuromuscular blocking agents are used in the protocols, monitoring of the neuromuscular blockade is mandatory, including measuring arterial blood pressure and neuromuscular blockade assessment. Possible complications include incomplete recovery from non-depolarizing neuromuscular blocking agents (postoperative residual curarization) and upper airway obstruction. Mechanomyography and acceleromyography techniques are the most used methods for neuromuscular blockade monitoring. Acceleromyography, due to its ease of use for research purposes, was presented in several studies involving pigs [86,87]. 

During the recovery phase from inhalation anesthesia, diligent and frequent monitoring is imperative, as life-threatening complications can arise [41]. Hypotension with mean arterial pressures less than 65 mmHg or systolic arterial pressures less than or equal to 85 mmHg is common in miniature pigs and may need intervention with dopamine or dobutamine (1–10 mg/kg/min continuous rate IV infusion for either), colloids, or fluid support [88]. 

When sedating pigs, respiratory obstruction can be a major concern. Oxygen can be supplied via the anesthesia machine or an oxygen demand valve, ideally with the pig placed in a sternal position [22]. Dorsal soft palate displacement, leading to airway obstruction, can develop in nonintubated pigs during anesthesia or after extubation [88,89]. One study on the majority of anesthesia-related complications during experimental invasive surgical procedures on pigs showed that, within the group of individuals at high anesthetic risk for invasive surgical operation, complications occurred in 20.31% of cases [12]. The majority of anesthetic difficulties involved intubation (14.06%), which led to the adjustment of the anesthetic approach by performing an emergency tracheotomy (6.25%) and keeping the anesthesia through an endotracheal tube attached to this level [12]. These types of complications need immediate attention and medical stabilization, as they can become life-threatening. In a liver injury model in pigs, vasopressin, as opposed to fluid resuscitation or saline placebo, resulted in prolonged survival and complete recovery from uncontrolled and otherwise fatal hemorrhagic shock [90]. Some complications may appear in correlation with the conditions in which the pigs are housed. Consequently, care should be used for any possible material to be ingested that can determine gastrointestinal foreign body blockages [88]. Limiting the number of pigs in stalls is important because bite wounds are common complications and can be a source of infection for experiments that involve surgical management [91].

Malignant hyperthermia (MH) is a disorder of skeletal muscle that starts as a hypermetabolic response that can be triggered in susceptible pigs by stress, a warm environment, volatile anesthetic gases, and the muscle relaxant succinylcholine [92]. Porcine stress syndrome and malignant hyperthermia can develop in genetically susceptible pigs when they interact with stressors, such as exertion, heat, or social interaction, or when they are exposed to certain medications or anesthetics that stimulate skeletal muscle [93]. MH affects humans, horses, dogs, and certain pig breeds and can be clinically manifested by hyperthermia, tachycardia, tachypnea, increased carbon dioxide production, increased oxygen consumption, acidosis, hyperkalemia, muscle rigidity, and rhabdomyolysis [63]. Halothane is traditionally considered the most likely volatile inhalant to trigger MH, but delayed onset of MH can also occur with exposure to isoflurane and desflurane [41]. Rhabdomyolysis is not a classic symptom of MH, but it can occur as a late complication during MH when muscle tissue breaks down and releases potassium and myoglobin into the bloodstream [92]. The effectiveness of injecting azumolene into pigs susceptible to MH is not fully understood but, as an analog of dantrolene (which is currently the only drug used to treat MH), azumolene is effective in reversing MH crisis in pigs in some studies [94,95]. A nanocrystalline dantrolene sodium suspension is also described as effective in the treatment of malignant hyperthermia and comparable to that of standard dantrolene sodium in pigs [96], but more research is needed to confirm its efficacy and safety.

## 8. Recovery

Proper post-anesthesia care, in a calm environment with the pig positioned in a sternal recumbency as soon as possible, is essential during recovery to ensure that the pig wakes up safely and without complications. It is advisable to retain the endotracheal tube until the pig begins moving its head spontaneously or can no longer tolerate the tube. Ideally, the pig should be placed with the head elevated and the neck extended to help maintain a patent airway [41]. Continuous monitoring of vital signs, which include heart rate, respiratory rate, body temperature, and oxygen saturation, is crucial during the recovery period and should be assessed for all major procedures at least every 15 min during recovery as it regains consciousness [91]. It is advisable to be ready to take action in the event of complications or any adverse reactions to anesthesia. Maintaining a warm and controlled environment to prevent the pig from getting too cold is essential, as pigs are susceptible to hypothermia during anesthesia and recovery. Mild hypothermia improved survival in a clinically relevant pig model of hemorrhagic shock and trauma [97]. Pain should be assessed and managed appropriately during recovery. The recovery area should be kept quiet and free from unnecessary disturbances, allowing a gradual and safe recovery.

## 9. Conclusions

Pigs share many anatomical and physiological similarities with humans, allowing extensive surgical procedures and monitoring, making them suitable for complex experiments. Proper anesthesia management is essential when conducting experiments involving animals and researchers must acquire a thorough knowledge of the techniques and protocols to be conducted [98]. Anesthesia is essential to minimize pain and distress in research animals. Pig anesthesia safeguards animal welfare, enables accurate data collection, facilitates standardized experiments, ensures the safety of both animals and researchers, and supports the development and validation of medical interventions. Pigs offer a level of consistency and reproducibility in experiments that may be more challenging to achieve with smaller animals. Researchers must adhere to strict ethical guidelines and obtain appropriate approvals. Continuing education and research procedures in terms of the Three Rs (replacement; reduction; refinement) are needed to ensure minimal use of pigs in research, along with a maximized welfare [99].

## Figures and Tables

**Table 1:** Premedication and sedative drugs used in pigs.

Agent	Dose	Route	Considerations, References
Azaperone	1–8 mg/kg (2–5 mg/kg mean)	IM	20 min to effect, sedative [21]
Acepromazine	0.03–1.1 mg/kg	IM, IV	tranquilizer [21,22]
Alfaxalone	5 mg/kg	IM	sedation [23,24]
Diazepam	0.2–1 mg/kg	IV	mild sedative [21,24]
Midazolam	0.1–0.5 mg/kg	IM, IV	sedation [21,24]
Xylazine	1–2 mg/kg	IM, IV	pigs are the least sensitive to xylazine [11]
Medetomidine	0.03–0.08 mg/kg	IM, IV	sedation and muscle relaxation [11,22]
Ketamine	2–30 mg/kg	IM, IV	poor muscle relaxation and analgesia [21,24,25]
Buprenorphine	0.01–0.05 mg/kg q 8–12 h.	IM, SC	significant respiratory depression [14,26]
Butorphanol	0.1–0.3 mg/kg q 4–6 h.	IM, IV	analgesia, short duration [21,25]
Tiletamine/Zolazepam Telazol^®^	2–8.8 mg/kg	IM, IV	sedation or anesthesia for minor surgery, 20–30 min, reversed with flumazenil 0.08 mg/kg [23]
Naloxone	0.5–2 mg/kg	IV	[21]
Glycopyrrolate	0.005–0.01 mg/kg	IM, IV	correct bradycardia, decrease salivation [9,15]
Atropine	0.02–0.04 mg/kg	IM, IV	correct bradycardia, decrease salivation [9,26]
**Combinations**			
Azaperone Midazolam	4 mg/kg azaperone	IM	[27,28]
	1 mg/kg midazolam		
Azaperone Xylazine	2 mg/kg azaperone	IM	[27,28]
	2 mg/kg xylazine		
Azaperone Butorphanol Ketamine	5 mg azaperone,	IM	[28,29]
	0.2 mg butorphanol		
	15 mg ketamine		
Azaperone Xylazine Ketamine	6 mg/kg azaperone	IM	[28,30]
	2 mg/kg xylazine		
	15 mg/kg ketamine		
Azaperone Midazolam Ketamine	2 mg/kg azaperone	IM	[21,28]
	0.3 mg/kg midazolam		
	15 mg/kg ketamine		
Acepromazine Ketamine	1.1 mg/kg acepromazine	IM	[21]
	33 mg/kg ketamine		
Alfaxalone Butorphanol Medetomidine	4 mg/kg alfaxalone	IM	[31]
	0.4 mg/kg butorphanol		
	40 μg/kg medetomidine		
Dexmedetomidine Ketamine Methadone	10 μg/kg dexmedetomidine	IM	Premedication, facilitate intubation [32]
	10 mg/kg ketamine		
	0.25–0.4 mg/kg methadone		
Xylazine Ketamine	1–2 mg/kg xylazine	IM	Premedication, short-term anesthesia [12,33]
	10–20 mg/kg ketamine		
Medetomidine Ketamine	0.04–08 mg/kg medetomidine	IV, IM	Premedication, short-term anesthesia [34]
	10 mg/kg ketamine		
	10 mg/kg ketamine		

**Table 2:** Induction agents in pigs.

Agent	Dose	Route	Considerations, References
Propofol	2–5 mg/kg	IV	[37,68]
Propofol Fentanyl	2 mg/kg 5 µg/kg	IV	allows intubation [14,46]
Dexmedetomidine Propofol	20–40 µg/kg dexmedetomidine 2–4 mg/kg propofol		[46]
Propofol Ketamine	1–1.5 mg/kg propofol 0.5–1 mg/kg ketamine	IV	sedation, induction, no respiratory depression, good recovery [68,69]
Alfaxalone	0.6–1.1 mg/kg	IV, IM	[46]
Etomidate	2–4 mg/kg	IV	provides cardiovascular stability [46,69]
Thiopental	10–20 mg/kg	IV	apnea, prolonged recovery [9]

## Data Availability

Not applicable.
